# Potassium Chloride as an Effective Alternative to Sodium Chloride in Delaying the Thermal Aggregation of Liquid Whole Egg

**DOI:** 10.3390/foods13071107

**Published:** 2024-04-04

**Authors:** Jiayu Guo, Xin Gao, Yujie Chi, Yuan Chi

**Affiliations:** 1 College of Food Science, Northeast Agricultural University, Harbin 150030, China; 15248099441@163.com (J.G.); gaoxin0427@163.com (X.G.); 2 College of Engineering, Northeast Agricultural University, Harbin 150030, China

**Keywords:** liquid whole egg, potassium chloride, aggregation behavior, rheological properties

## Abstract

The potential of potassium chloride (KCl) to be used as a substitute for sodium chloride (NaCl) was studied by monitoring the effects of salt treatment on thermal behavior, aggregation kinetics, rheological properties, and protein conformational changes. The results show that the addition of KCl can improve solubility, reduce turbidity and particle size, and positively influence rheological parameters such as apparent viscosity, consistency coefficient (*K* value), and fluidity index (*n*). These changes indicate delayed thermal denaturation. In addition, KCl decreased the content of β-sheet and random coil structures and increased the content of α-helix and β-turn structures. The optimal results were obtained with 2% KCl addition, leading to an increase in T_p_ up to 85.09 °C. The correlation results showed that T_p_ was positively correlated with solubility, α-helix and β-turn but negatively correlated with Δ*H*, turbidity, β-sheet and random coil. Overall, compared to NaCl, 2% KCl is more effective in delaying the thermal aggregation of LWE, and these findings lay a solid theoretical foundation for the study of sodium substitutes in heat-resistant liquid egg products.

## 1. Introduction

Liquid whole egg (LWE) is a valuable raw material for the production of high value-added products, offering advantages in terms of transportation, storage, and food safety compared to traditional eggs. However, the heat sensitivity of LWE poses challenges due to protein structure destruction and denaturation, resulting in gel formation through cross-linking when LWE is heated beyond 64.5 °C. Moreover, these processes lead to increased turbidity and viscosity while decreasing solubility [1]. Industrial production guidelines typically mandate a maximum protein solubility loss of 5%. However, this requirement is incompatible with the increasing demand for more stringent heat treatments since LWE produced under these conditions will not be efficiently sterilized. These conflicting factors limit the opportunities for advancing liquid egg products, which has prompted egg companies worldwide to prioritize enhancing the thermal stability of LWE. This issue currently represents a prominent area of research.

Currently, enhancing heat resistance is primarily achieved by incorporating sodium salt. Chalamaiah et al. [2] reported that NaCl (0.5 and 1 M) significantly affected protein solubility. Under normal circumstances, an increase in solubility can effectively reduce the repulsive interaction between adsorbed and unadsorbed proteins, resulting in prolonged gelation time. Consequently, the overall thermal resistance of LWE increases, while aggregation decreases. NaCl, an exceptionally soluble common salt, strongly influences the texture, shelf life, and characteristics of food. Primacella et al. [3] reported that with the addition of salt, the rheological properties of liquid eggs are similar to those of fresh eggs. However, excessive use of high-sodium salt may lead to various health complications, including cardiovascular, cerebrovascular, kidney and stroke diseases [4]. Therefore, it is imperative to identify alternative substances that can replace NaCl to reduce the thermosensitivity of LWE.

KCl and NaCl are both monovalent chloride salts that have similar effects. KCl is commonly used as a substitute for sodium salt. Research has demonstrated a significant positive relationship between higher potassium consumption and a reduced risk of coronary heart disease and stroke. Conversely, insufficient potassium intake and excessive sodium consumption have been linked to the development of cardiovascular disease [5]. To mitigate the risk of hypertension, cardiovascular disease, stroke, and coronary heart disease, the World Health Organization recommends that adults increase their daily potassium intake to 3.5 g [6]. Adequate potassium consumption is also crucial for maintaining heart and bone health. In the realm of food processing, the utilization of chloride salts like KCl, CaCl_2_, and MgCl_2_ as substitutes for NaCl has been shown to be an effective and safe approach to inhibit the growth of microorganisms in salted meat products [7]. Wang et al. [8] investigated the partial substitution of KCl in pickled salted eggs. The findings demonstrated that both the Na^+^ group and the Na^+^ + K^+^ group exhibited similar reductions in viscosity. Additionally, the effective inhibition of sodium penetration in salted eggs resulted in a decrease in sodium content without compromising sensory quality. Ions help regulate the concentration and stability of crucial gel proteins. The introduction of small amounts of NaCl or KCl facilitated rapid flow in an egg white powder (EWP) gel with a protein concentration of 15 mg/mL [9]. The impact of NaCl on particle structure and water dispersion at pH values of 2.0 to 10.0 was investigated by Li et al. [10], who found that NaCl exhibited a Janus effect. Additionally, they conducted experiments using KCl and CsCl and observed no significant difference in dispersion at equivalent salt concentrations. This similarity implies that the effects of K^+^ and Cs^+^ on disrupting calcium bridges between egg yolk particles are comparable to those of NaCl. These findings underscore the potential of KCl for producing low-sodium products and offer valuable insights for the future use of potassium salts as substitutes for sodium salts in the food industry.

To the best of our knowledge, limited information is available regarding the replacement of NaCl with other salts to enhance the thermal stability of LWE. The objective of this study was to address this knowledge gap and develop strategies for reducing the sodium content and enhancing the thermal stability of LWE. We investigated the possibility of improving thermal stability by substituting NaCl with KCl, focusing on the effects on the thermal aggregation of LWE.

## 2. Materials and Methods

### 2.1. Materials and Reagents

Fresh shelled eggs (average weight 45~55 g) were obtained from Shuangcheng County Farm, Heilongjiang Province, China. Sodium chloride (NaCl) was purchased from Beijing China Salt Co. Potassium chloride (KCl) was obtained from Jiangsu Lianyungang Corde Food Ingredients Co., Ltd. (Lianyungang, China) Phosphate buffer solution (PBS, 10 mM, pH 7.2) and Coomassie brilliant blue G250 were obtained from Beijing Solarbio Technology Co., Ltd. (Beijing, China). All reagents used were of analytical grade.

### 2.2. Samples Elaboration

#### 2.2.1. Preparation of LWE

A fresh egg with an intact eggshell was carefully washed, dried, and cracked. All chalaza was meticulously separated from the whole egg using tweezers. The liquid content of each whole egg was collected in a beaker and then whisked thoroughly to ensure even blending. After allowing it to stand for defoaming, any insoluble matter was sifted out. In the experimental group, KCl-treated samples (LWE-K) and NaCl-treated samples (LWE-Na) were prepared by adding salt solutions (KCl or NaCl) with varying concentrations of ions (1%, 2%, 4%, or 6%); in the control group (LWE), no salt was added. All samples were gently stirred using a magnetic stirrer until homogenized for further application. All procedures were conducted at room temperature (approximately 25 °C).

#### 2.2.2. Heat Treatment of LWE Samples

The samples were subjected to high-temperature sterilization (76 °C for 3 min), followed by immediate transfer and rapid cooling in ice water for 1 min.

### 2.3. Samples Evaluation

#### 2.3.1. Color Characterization

Sample color was determined according to the method in [11] to determine the effect of salt treatment on the macroscopic state of LWE. A portable spectrophotometer (CM-700 d, KONICA MINOLTA Ltd., Tokyo, Japan) was used for color characterization of LWE with varying salt concentrations. The experiment was performed in triplicate at various locations on a flat dish. The chromatic values of L* (lightness), a* (redness), and b* (yellowness) were quantified, while *dE* was used to quantify the magnitude of color variation and was calculated as follows (1):

(1) $$dE=\sqrt{{\left(dL^{*}\right)}^{2}+{\left({da}^{*}\right)}^{2}+{\left(db^{*}\right)}^{2}}$$

where L* represents the lightness on a 0–100 scale from black to white, a* denotes the scale of red (+) or green (−), and b* denotes the scale of yellow (+) or blue (−).

#### 2.3.2. Solubility

A multifunctional microplate reader (SpectraMax reg iD3, Meigu Instrument Co., Shanghai, China) was used to determine the solubility of LWE at different salt concentrations to assess the colloidal stability of LWE protein. Diluted salted LWE samples were centrifuged at 10,000× *g* for 20 min. The resulting supernatants were collected, and the protein contents were quantified by the Bradford method [12]. Protein solubility (%) is expressed using the following formula:

(2) $$Solubility(\%)=\frac{C_{1}}{C_{0}}\times 100$$

where *C*_0_ and *C*_1_ are the protein concentrations before and after centrifugation, respectively.

#### 2.3.3. Turbidity

The LWE samples were diluted using 10 mmol/L pH 7.2 PBS. The transmittance (T%) was measured at a wavelength of 600 nm using a UV spectrophotometer (UV-1800, Shimadzu Co., Kyoto, Japan) to assess the turbidity of the LWE samples. PBS served as the blank control and had a transmittance value of 1 [13].

(3) $$Turbidity\left({OD}_{600nm}\right)=-\ln\left(T\%\right)$$

where T% is the transmittance of the sample, representing the percentage of incident light that is transmitted through the solution. A transmittance value of 100% indicates that the solution is transparent and exhibits no turbidity.

#### 2.3.4. Particle Size Distribution (PSD)

The particle size distribution (PSD) was determined according to the method described by Li et al. [14] with minor modifications. A laser particle analyzer (S3500, Microtrac Inc., Largo, FL, USA) was used to determine the PSD of the samples and thus provide insight into the level of protein aggregation in LWE. The samples were diluted before measurement. The refractive index of the sample was set to 1.46, the refractive index of water was set to 1.33, and the temperature was maintained at 25 °C ± 2 °C.

#### 2.3.5. Rheological Properties

##### Apparent Viscosity

A rotating module rheometer (HAAKE MARS60, Thermo Fisher Scientific Inc., Waltham, MA, USA) was used to determine the apparent viscosity of the samples. A continuous scanning program in CS/CR mode was used to observe the changes in viscosity at shear rates from 0 to 100 s^−1^ to characterize the rheological properties of the liquid. A 35 mm plate–plate configuration was used to test the samples at a gap distance of 0.05 mm and a temperature of 25 °C. Three replicate tests were conducted for each sample, and a power law model was utilized to fit the flow data [15]. The following equation was applied for calculating the flow curves.

(4) $$\eta =K\cdot \gamma^{n-1}$$

where *η* represents the apparent viscosity (Pa), *K* represents the consistency index (Pa·s^n^), *γ* represents the shear rate (s^−1^), and *n* represents the flow behavior index.

##### Temperature Sweep

Temperature sweep measurements were performed using a rotational rheometer (HAAKE MARS60, Thermo Fisher Scientific Inc., Waltham, MA, USA). The thermal transition temperature was determined by measuring the storage modulus (G′) of the samples using temperature sweep mode [16]. The rheometer was equipped with a plate/plate measurement system (diameter = 35 mm). To prevent water evaporation from the sample, a silicone oil seal was applied between the two plates. Prior to initiating the temperature ramp, the sample was preheated at 25 °C for 1 min on the plate. The temperature ramp ranged from 25 °C to 90 °C with a heating rate of 5 °C/min. The thermal transition temperature of each sample was determined as the temperature at which the inflection point of G′ occurred.

#### 2.3.6. Differential Scanning Calorimetry (DSC)

The heat flow curves of LWE samples with different concentrations of KCl and NaCl were recorded using a differential scanning calorimeter (DSC 2500, TA Instruments Co., New Castle, DE, USA) to determine the thermal properties of the samples. The LWE samples were weighed (10.0–15.0 mg), placed in an aluminum crucible, and sealed with a tablet press. The sample was heated from 25 to 105 °C at a heating rate of 10 °C/min under a nitrogen flow rate of 50 mL/min. Good contact was ensured between the sample and the bottom of the aluminum pan. Thermograms were analyzed with Universal Analysis Software (Version 4.5A, TA Instruments), which directly reports the onset temperature (*T*_onset_, °C), the thermal denaturation peak temperature (*T*_p_), the endset temperature (*T*_endset_, °C) and the enthalpy of denaturation (Δ*H*, J/g) [17].

#### 2.3.7. Secondary Structure

LWE samples with various concentrations of KCl and NaCl were analyzed using Fourier transform infrared (FTIR) spectroscopy (TENSOR II, Bruker Co., Karlsruhe, Germany) [18]. The FTIR spectrum was recorded in the wavenumber range of 400–4000 cm^−1^ with a resolution of 4 cm^−1^ over 32 scans. To more comprehensively monitor changes in protein secondary structure, we employed Peakfit 4.12 software (Thermo Electron Co., Waltham, MA, USA) and OMNIC 9.0 software (Nicolet Co., Nicollet, MN, USA). The amide I band (1600–1700 cm^−1^) was analyzed via deconvolution, second-order derivative processing, and curve-fitting analysis to accurately determine the secondary structure of the samples. Specifically, the amide I region was assigned as follows: β-sheet (1600–1640 cm^−1^), random coil (1640–1650 cm^−1^), α-helix (1650–1660 cm^−1^), and β-turn (1660–1700 cm^−1^). Each sample was tested in triplicate.

#### 2.3.8. Tertiary Structure

We used a fluorescence spectrophotometer (F-7100, Hitachi Co., Tokyo, Japan) to perform fluorescence intensity scanning to analyze the tertiary structure of the LWE samples. Each sample was diluted to a concentration of 0.1–0.5 mg/mL using PBS (10 mmol/L, pH 7.2). Endogenous fluorescence measurements were performed utilizing an excitation wavelength of 280 nm, a scanning range from 300 nm to 450 nm, and a slit width of 5 nm [19].

#### 2.3.9. Surface Hydrophobicity

The fluorescence intensity of the samples was measured using a fluorescence spectrophotometer (F-7100, Hitachi Co., Tokyo, Japan). The protein solution was diluted to a concentration of 0.1–0.5 mg/mL and mixed thoroughly with 20 μL of 8-anilino-1-naphthalenesulfonic acid (ANS) solution (8 mM ANS dissolved in 0.1 M potassium phosphate buffer, pH 7.0). The reaction proceeded at room temperature in darkness for one hour, with excitation at a wavelength of 350 nm and scanning from 420 nm to 670 nm. A slit width of 5 nm was used to measure the fluorescence intensity. The obtained maximum fluorescence intensity was used to assess the surface hydrophobicity of the LWE samples [20].

#### 2.3.10. Statistical Analysis

Statistical analyses were conducted by using SPSS 22 statistics software (SPSS Inc., Chicago, IL, USA) with the least significant difference (LSD) and Duncan’s comparison tests. Pearson’s test was used to evaluate the correlations between the physical and chemical properties, thermal properties, and structural properties of the LWE samples. Differences were considered statistically significant at *p* < 0.05. All figures were generated using Origin 2024 (OriginLab Co., Northampton, MA, USA). All experiments were conducted in triplicate, and the results are presented as the mean ± standard deviation.

## 3. Results and Discussion

### 3.1. Effects of KCl and NaCl on the Appearance and Color of LWEs

**Figure 1:**
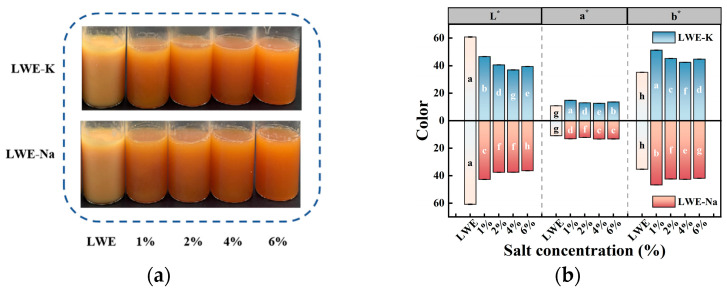
Effects of KCl and NaCl on the macroscopic characteristics of LWE: (**a**) visual appearance and (**b**) color characteristics. Different letters indicate significant differences (*p* < 0.05) among the data within each group.

The color of LWE is a crucial factor determining the acceptability of the product. As shown in Figure 1, the addition of salt significantly improved the flow properties of heat-treated LWE, and compared with those in the control group, the colors of the samples with added salt were darker, redder, and more yellow (*p* < 0.05).

Analysis of the influence of different salts and concentrations on color parameters revealed that, compared with the control group, the addition of KCl resulted in an increase in a* and b* values, while the L* value decreased (*p* < 0.05). The addition of different concentrations of KCl and NaCl had similar effects on color parameters; however, the color parameters of the KCl treatment group tended to first decrease and then increase with increasing KCl concentration. This could be attributed to an increase in conjugated groups, as the color of LWE is primarily determined by carotenoids with long-chain conjugated carbon double bonds [21]. The introduction of KCl and NaCl may cause Cl^−^ and carbonyl groups to combine with carotenoids, shifting the chromophore absorption peak toward longer wavelengths and thereby improving the color characteristics of LWE [22]. The results suggest that KCl and NaCl have comparable macroscopic influences on the state and color of LWE.

### 3.2. Effects of KCl and NaCl on LWE Aggregation Behavior

**Figure 2:**
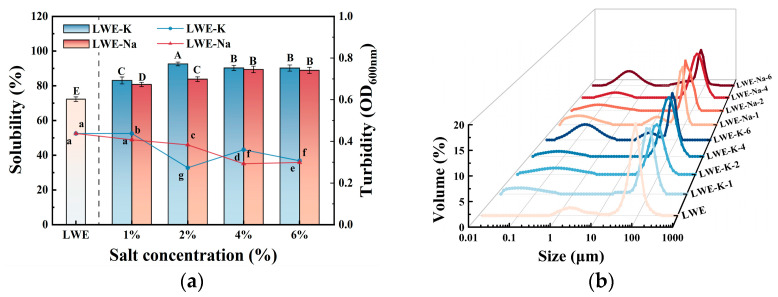
Effects of KCl and NaCl on the aggregation behavior of LWE: (**a**) solubility and turbidity and (**b**) particle size distribution. Different letters indicate significant differences (*p* < 0.05) among the data within each group.

As shown in Figure 2, the effect of salt addition on heat aggregation behavior was characterized by measuring the solubility, turbidity and PSD of LWE solutions. Solubility is an essential parameter for assessing LWE denaturation [23], while turbidity indicates the extent of protein aggregation. Previous research has demonstrated a negative correlation between turbidity and solubility, as insoluble mixtures exhibit high levels of turbidity due to light scattering caused by insoluble particles [24]. PSD is a critical parameter that reflects conformational changes, effectively indicating the cross-linking and aggregation of proteins. The addition of chlorine salts has positive effects on the solubility, turbidity and PSD of LWE.

Compared to the control, the solubility of the KCl treatments exhibited an overall improvement, and the influence of KCl was greater than that of NaCl at the same concentration. Solubility initially increased and then decreased with increasing salt concentration. Notably, the solubility of LWE significantly increased by 28.0% after the addition of 2% KCl (*p* < 0.05). This may be because the presence of KCl or NaCl induces an ionic environment [25], thereby enhancing the negative net charge and electrostatic repulsion of proteins. These enhancements promote protein solvation and effectively inhibit heat-induced aggregation and insoluble aggregate formation. Consequently, higher temperatures are required for aggregation and network structure formation, and heat resistance is improved [15,26,27]. However, at high salt concentrations, solubility decreases due to the salting-out effect, which impacts hydrophobic interactions. Strong hydrophobic interactions promote an increase in β-sheet structures between proteins, resulting in enhanced cohesion among protein molecules [28,29].

Compared to the control group, the turbidity significantly decreased when different concentrations of KCl and NaCl were added, with the exception of the 1% KCl treatment (*p* < 0.05). Even at 76 °C, the LWE samples remained transparent after being heated for 3 min. The lowest turbidity was achieved at a KCl concentration of 2% (*p* < 0.05). This can be attributed to the enhanced interactions between negatively charged Cl^−^ ions and positively charged protein regions, which effectively inhibited protein aggregation during heating and subsequently reduced the turbidity of the mixture. At a concentration of 2%, the improvement effect of KCl was greater than that of NaCl, which can be attributed to the increase in continuous phase viscosity induced by KCl, resulting in reduced collisions between proteins and slower protein gelation [30]. The inhibitory effect of KCl may also arise from its partial interactions with hydrophobic surface regions, which prevent protein unfolding and stabilize aggregation-prone intermediates. However, at high salt concentrations, the surface tension of the solution increases, leading to salting out and subsequent precipitation of protein molecules [26]. Additional analyses are required to confirm the aggregation behavior of LWE. This can be accomplished through particle size analysis and the assessment of other relevant indicators.

The samples exhibited a bimodal distribution, with the primary peak encompassing large particles from 10–1000 μm. With the addition of KCl, the PSD became more dispersed compared to the control. This is consistent with the previous turbidity findings, suggesting a decrease in protein aggregation and an increase in protein solubility. Additionally, the subpeak corresponding to 1–10 μm particles shifted left, accompanied by the emergence of a new peak in the range of 0.01–1 μm. At low concentrations, the KCl-treated samples exhibited an increased number of small particles 0.01–1 μm in diameter compared to the NaCl-treated samples. This result suggested that the addition of KCl and NaCl results in the substitution of Ca^2+^ ions in calcium bridges by Na^+^ and K^+^, leading to particle dissociation. Additionally, potassium ions exhibit a greater capacity than sodium ions for reducing particle size [10,29,31]. The curves for the high-concentration KCl-treated samples displayed a trimodal distribution, with peaks in the ranges of 0.1–10 μm, 10–100 μm and 100–1000 μm. In comparison to the control, the main peak was smaller, and the distribution was more dispersed, with an increased proportion of smaller particles. This difference suggested that salt not only influences aggregation behavior but also induces the disruption and rearrangement of lipoproteins, resulting in increased friction within the system. This increase in friction also accounts for the abrupt increase in viscosity [32,33]. Additionally, the peak in the 0.1–10 μm range became broader, accompanied by the emergence of a distinct peak in the 10–100 μm range. This new peak indicated the progressive aggregation of smaller particles as well as the potential for further coalescence through intermolecular forces [34]. Salts increase electronegativity, facilitating protein aggregation and resulting in an increase in particle size. As the concentration increased, the main peak shifted to the right, indicating the reaggregation of dissociated particles. The impact of KCl at low and high concentrations on the PSD of heat-treated LWEs was similar to that of NaCl. Moreover, the primary peak in the PSD was narrower under low-salt conditions, indicating a stronger inhibitory effect on protein aggregation [35].

Overall, the addition of KCl has been demonstrated to effectively inhibit protein unfolding and aggregation, thereby exerting a significant influence on the aggregation behavior of LWEs. This effect is achieved through improvements in turbidity, solubility, and PSD, ultimately leading to enhanced thermal stability. Moreover, a larger aggregate size can lead to increased internal friction, while variations in aggregate size can account for the observed variability in rheological properties [36].

### 3.3. Rheological Analysis: Apparent Viscosity and Temperature

#### 3.3.1. Apparent Viscosity

**Figure 3:**
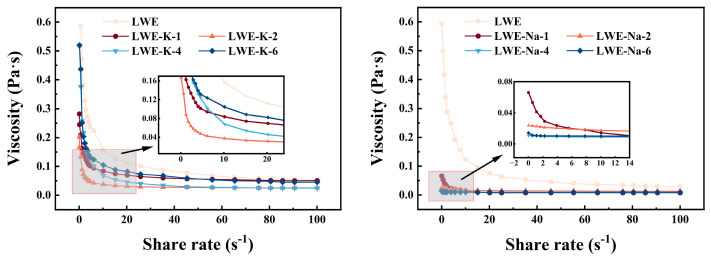
Effects of KCl and NaCl on the apparent viscosity of LWEs.

The apparent viscosity is a crucial parameter for assessing the denaturation and aggregation behavior of proteins and for investigating protein–protein interactions on the basis of the relationship between shear rate and viscosity. As shown in Figure 3, rheological measurements revealed consistent flow curves across the entire range of shear rates for both the control group and the salt-treated group. At low shear rates, the apparent viscosity decreased, followed by stabilization at high shear rates, as shown in Table 1. The consistency index (*K*) is directly proportional to the viscosity.

These findings indicate a significant disruption in the network structure of LWE, resulting in the degradation of protein orientation, arrangement, and overall structure. Consequently, LWE exhibits Newtonian fluid-like characteristics [37,38]. These characteristics can be attributed to the decrease in intermolecular entanglement of cross-linked molecules resulting from the disruption of weak protein bonds at higher shear rates. In the control group, the initial apparent viscosity was high, indicating that heating enhanced the apparent viscosity while diminishing shear-thinning behavior. This effect can be attributed to protein denaturation, wherein active groups on protein molecules become exposed, leading to protein aggregation through hydrophobic interactions. Moreover, denatured protein molecules interact with other proteins, resulting in elongation and the formation of larger particles [22,39]. Compared to the control, the addition of KCl induced an overall downward shift in the rheological curve, leading to a significant reduction in the viscosity of the salted LWE samples within each experimental group (*p* < 0.05). The findings suggest that salt protects proteins by inhibiting their denaturation and polymerization, thereby impeding the gradual upward movement of the flow curve under heat treatment and ultimately leading to enhanced thermotolerance. Additionally, the enhancement effect of KCl on viscosity was similar to that of NaCl, potentially due to the monovalent nature of both K^+^ and Na^+^ ions [40]. On the one hand, salt interacts with proteins, particularly dense proteins, by forming bonds between K^+^ and protein carboxyl groups and between Cl^−^ and protein amino groups. This binding behavior amplifies hydrophobic interactions between protein molecules, leading to reduced LWE viscosity [41]. On the other hand, ion-induced electrostatic shielding impedes water molecule–protein interactions involving hydrophilic charged groups, thereby preventing protein aggregation and better preserving the native protein conformation [28,42]. The addition of 2% KCl resulted in the most pronounced decrease in the flow curve, which is consistent with the LWE aggregation behavior (*p* < 0.05). This effect can be attributed to the ionic environment created by KCl, which leads to changes in osmotic pressure and enhances hydration, ultimately stabilizing protein structure and inhibiting protein aggregation. However, the viscosity increased at high salt concentrations. This increase can be attributed to the induction of protein aggregation through electrostatic interactions caused by elevated salt concentrations, which facilitated the formation of complexes in LWE [43].

The *K* value of the control group was above 0.490 ± 0.004 Pa·s, and the value decreased when KCl was added (*p* < 0.05), consistent with the viscosity results. This effect can be attributed to the exposure of active groups within protein molecules during heat treatment. This exposure accelerates lipoprotein aggregation through hydrophobic interactions, leading to an increase in *K*. However, the addition of salt can inhibit aggregation, resulting in a decrease in *K*. Moreover, with increasing salt concentration, the *K* decreases further. Previous studies have demonstrated a positive correlation between *K* value and temperature [32], confirming that KCl can effectively impede the thermal aggregation of LWEs and mitigate the increase in viscosity induced by high temperatures. The flow behavior index *n* indicates the degree to which a fluid differs from a Newtonian fluid. According to our observations, the *n* value ranged from 0.520 ± 0.007 to 0.756 ± 0.009 Pa·s, indicating the occurrence of shear-thinning behavior across the range of tested shear rates (0.1–100 s^−1^). This trend implies that the addition of KCl or NaCl does not substantially change the characteristic non-Newtonian rheological properties of LWE [44]. The *n* value generally exhibits an inverse correlation with fluidity. Heating decreases the fluidity of LWE, while the addition of salt can effectively retard protein denaturation, enhance heat resistance, and decelerate protein recombination and polymerization, thereby mitigating the reduction in *n* [36].

#### 3.3.2. Temperature Scan

**Figure 4:**
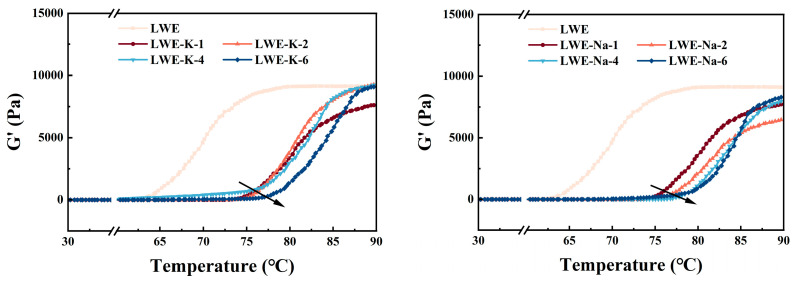
Effects of KCl and NaCl on the temperature scanning results of LWEs.

The variation in the storage modulus G′ with temperature was monitored to analyze aggregation and denaturation in the LWE samples. As depicted in Figure 4, the rheological behavior of all the samples demonstrated a distinct three-stage pattern.

With increasing temperature, G′ steadily increased, followed by a sharp increase after the inflection point and eventually reaching a plateau [35]. The inflection point on the temperature curve indicates the thermal transition temperature of protein. In this study, the LWE thermal transition temperature was 64.81 °C, while the LWEs containing 1%, 2%, 4%, and 6% KCl had thermal transition temperatures of 76.38, 76.76, 79.34, and 80.63 °C (*p* < 0.05), respectively. This finding implies that salt can effectively retard the denaturation kinetics of LWE. This delay can be attributed to the balance between the inhibition of protein unfolding and promotion of protein–protein interactions, which ultimately determines protein thermal stability. The addition of salt reduces the frequency of collisions between proteins and hinders protein unfolding. Moreover, salt shields repulsive forces among protein molecules, enhancing randomness in the aggregation and embedding of nonpolar groups and inhibiting aggregation. Consequently, salt induces disordered aggregation and elevates the temperature threshold for network formation throughout the entire system [45]. The salt concentration also affects the delay in the thermal transition temperature. A previous study demonstrated that incorporating 0.3% NaCl results in a delay of 50–70% in the thermal transition temperature. The concentrations of NaCl and KCl added in this study delayed the thermal transition temperature to 76–81 °C. This finding is consistent with the results of Li et al., who reported a delay to 75–80 °C with the addition of 3% NaCl [46]. These results provide further evidence that KCl has an equivalent impact to NaCl on reducing the heat sensitivity of LWE.

### 3.4. Effects of KCl and NaCl on the Differential Scanning Calorimetry (DSC) Results of LWE

**Figure 5:**
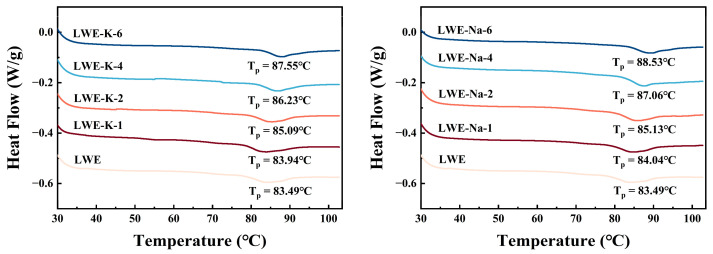
Effects of KCl and NaCl on the differential scanning calorimetry (DSC) results of LWE.

Differential scanning calorimetry (DSC) is widely used to analyze the thermal denaturation characteristics of food proteins, providing valuable insights into protein stability under heating. Protein denaturation typically involves a conformational change from ordered to disordered or from folded to unfolded [45]. To further understand the impact of KCl on thermal stability, the thermal solidification behaviors of both KCl and NaCl were analyzed. As depicted in Figure 5, the DSC curves of all the samples exhibited a consistent trend, characterized by the presence of an endothermic peak at 80–90 °C. As shown in Table 2, the onset temperature (*T*_onset_), thermal denaturation peak temperature (*T*_P_), endset temperature (*T*_endset_), and denaturation enthalpy (Δ*H*) were monitored during the heating process. The addition of salt generally resulted in an increase in both *T*_onset_ and *T*_P_ (*p* < 0.05).

Compared with the control, the endothermic peak of the samples treated with various salt solutions exhibited a rightward shift. *T*_p_ represents the thermal coagulation temperature of protein. A higher *T*_p_ indicates enhanced thermal stability [47]. Treatment with 6% KCl resulted in an increase in *T*_p_ to 87.55 ± 0.21 °C, which was greater than that of the control (*p* < 0.05). Moreover, the increase in *T*_p_ was directly proportional to the quantity of KCl added. These findings suggest that the addition of KCl can effectively enhance the thermal stability of LWE by mitigating denaturation and coagulation during heating. In the 2% KCl treatment group, the heat absorption peak was shallower. A steeper heat absorption peak indicates a higher Δ*H* and more pronounced conformational changes [48], suggesting that the treatment group had less total heat absorption during thermal denaturation and more stable conformational changes.

The addition of 6% KCl led to a 6.05% increase in *T*_onset_ and a 4.85% increase in *T*_P_ compared to the control group (*p* < 0.05). This result can be attributed to the effects of the added ions on protecting structural integrity, reinforcing hydrogen bonding between molecules, and impeding interactions with water molecules, thereby increasing the initial temperature of thermal solidification. However, the impact of salt treatment on the *T*_endset_ and Δ*H* of the LWE samples was found to be not statistically significant (*p* > 0.05). These findings suggest that KCl has a comparable effect to that of NaCl on enhancing the thermal stability of LWE. The influence of KCl and NaCl salts on the thermal properties of meat may involve the same mechanism [48]; this similarity may offer valuable insights for substituting KCl for NaCl during the processing of LWE products.

### 3.5. Effects of KCl and NaCl on the Secondary Structure of LWE

Fourier transform infrared (FTIR) spectroscopy, also known as total reflectance (TR)-FTIR spectroscopy, is a powerful technique for analyzing the microenvironment of side chains and protein structure at the molecular level. It has been successfully used to investigate changes in protein secondary structure during thermal denaturation. The complexity of infrared spectra generally arises from the specific hydrogen-bonding interactions involving OH, CN, CO, and NH groups in each polymorphic form. Proteins exhibit distinct infrared absorption bands; in particular, the amide I band (1600–1700 cm^−1^) and amide II band (1480–1575 cm^−1^) are primary bands for investigating protein secondary structure. Specifically, the amide I band is highly sensitive to structural changes [49,50].

**Figure 6:**
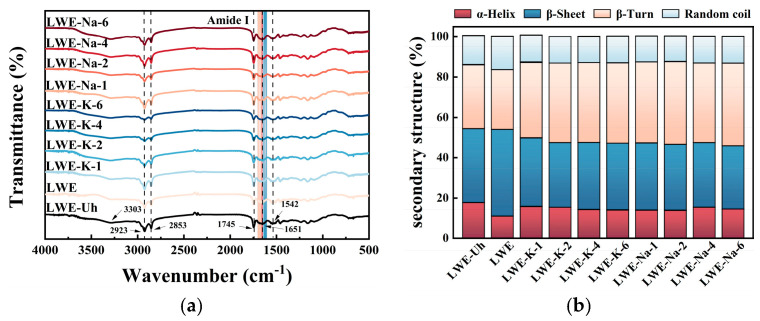
Effects of KCl and NaCl on the secondary structure of LWE: (**a**) FTIR spectra and (**b**) secondary structure.

As shown in Figure 6, the characteristics of unheated fresh liquid whole egg (LWE-Uh), fresh liquid whole egg (LWE), and liquid whole egg with varying concentrations of KCl and NaCl added (LWE-K and LWE-Na) were investigated using FTIR spectroscopy to elucidate the ion-induced changes in the secondary structure of LWEs following thermal treatment.

In all the samples, characteristic absorption bands were observed at wavenumbers of 3303 cm^−1^, 2923 cm^−1^, 2853 cm^−1^, 1745 cm^−1^, 1651 cm^−1^, and 1542 cm^−1^. The band at 3303 cm^−1^ corresponds to the stretching vibration of OH or NH, whereas the bands at 2923 cm^−1^, 2853 cm^−1^, and 1745 cm^−1^ represent the symmetric stretching vibrations of CH_2_, -CH_3_, and the ester bond (-CO), respectively. The band at 1651 cm^−1^ indicates the C=O stretching vibration of amide I, which is related to the α-helical structure and is susceptible to hydrogen bonding. Heat treatment of LWE without added salt resulted in the emergence of new narrow bands at 1645 cm^−1^, 1625 cm^−1^, and 1616 cm^−1^, indicating a conformational transition from α-helix to β-sheet and random coil structures. These findings provide evidence for the heat-induced aggregation of protein molecules, which is primarily attributed to the disruption of hydrogen bonds induced by thermal treatment. Aggregated proteins typically exhibit a distinct peak at or below 1620 cm^−1^, which is indicative of intermolecular aggregates. Furthermore, protein unfolding facilitates the formation of random coil structures. The addition of salt prior to heat treatment effectively preserved the native secondary structure of the proteins and prevented unfolding. The addition of different concentrations of KCl induced a redshift in the amide I band and a narrowing of the characteristic absorption band at approximately 1653 cm^−1^. These effects can be ascribed to the influence of Cl^−^ ions on the intensity of the constituent bands, leading to structural reorganization of the surrounding water molecules and an increase in protein order. This results in an increase in vibration frequency and decrease in half-width. Furthermore, salt addition induced a shift in the CO stretching band of amide I in heat-treated LWE to 1647 and 1684 cm^−1^, which is indicative of a disordered conformation (random coils and β-turns) [30]. The amide I band shifted to a higher position, indicating the weakening of hydrogen bonds, as previously reported. Thus, the reduction in protein aggregation and disruption of intermolecular hydrogen bonding caused by KCl have been experimentally confirmed. The FTIR spectra after the addition of KCl and NaCl exhibited comparable peak positions and trends, suggesting that the addition of salt does not significantly affect the chemical bonds and functional groups of proteins.

The protein secondary structure in the heat-treated LWE samples was analyzed by fitting curves to the CO and CN stretching bands in the amide I region (1600–1700 cm^−1^). In fresh LWE, the secondary structure comprised 17.77% α-helices, 36.50% β-sheets, 31.61% β-turns, and 14.13% random coils. α-Helices are stabilized by intramolecular hydrogen bonds between the carbonyl (-CO) oxygen atom and the amino (NH-) hydrogen atom of the polypeptide chain. This structural motif is buried within the protein and exhibits amphiphilic characteristics with both hydrophilic and hydrophobic regions, which contribute significantly to the stability of the apolipoprotein structure [19]. β-Sheet structures are typically stabilized by intermolecular hydrogen bonds, while β-turns are located on the surface of proteins, particularly globular proteins, and contain polar and charged amino acid residues. These residues contribute to the hydrophilicity of the protein [37]. The protein structure of the heat-treated LWEs significantly changed, characterized by a reduction in the α-helix and β-turn contents and an increase in the β-sheet and random coil contents (*p* < 0.05). This change can be ascribed to the partial unfolding and subsequent aggregation of the α-helical structure. The protein molecules unfold, promoting the formation of intermolecular hydrogen bonds and facilitating aggregation. The increased formation of intermolecular hydrogen bonds also contributes to the elevated β-sheet content [32]. Furthermore, the reduction in β-turn content implies a decrease in protein hydrophilicity, potentially due to the exposure of nonpolar amino acids. Heating facilitates the transition from β-turns to random coils, leading to the disruption of weak hydrogen bonds and protein denaturation [13,19]. The observed changes in protein structure and increased aggregation may be attributed to protein–lipid interactions. Additionally, changes in reactive groups can affect the stability and aggregation of lipoproteins. Compared to the control group, the addition of various concentrations of KCl to LWE resulted in a significant increase in the proportions of α-helix and β-turn structures following heat treatment. Conversely, there was a notable decrease in the percentages of β-sheet and random coil structures (*p* < 0.05). This finding implies that the addition of KCl to LWEs can effectively impede the conformational change from α-helix to β-sheet and random coil structures. These findings confirm that increased ionic strength reduces the prevalence of ordered secondary structures (β-sheets), reduces protein collisions and contacts, and hinders protein aggregation by decreasing hydrogen bonding interactions between proteins [45,51]. Additionally, studies have indicated that an increase in electrostatic repulsion between protein molecules may contribute to a decrease in random coil content and an increase in α-helix content. An increase in β-turn content and decrease in the random coil content indicate an increase in stability [13,46]. These changes are consistent with the observed inhibition of protein aggregation in LWE, providing further support for the findings related to aggregation state, flow properties, and thermal stability. Additionally, the observed changes in the secondary structure of LWE induced by KCl and NaCl were consistent with the FTIR spectroscopy results.

### 3.6. Effects of KCl and NaCl on the Tertiary Structure of LWE

#### 3.6.1. Endogenous Fluorescence Spectroscopy

**Figure 7:**
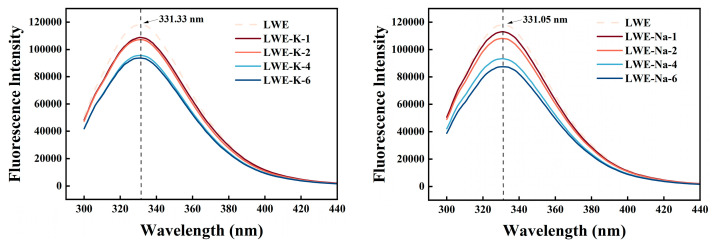
Effects of KCl and NaCl on the endogenous fluorescence spectrum of LWE.

Fluorescence spectroscopy is a powerful technique for the analysis of inherent fluorescent chromophores in biological macromolecules or chromophoric molecules added as labels. This method enables visual detection of changes in protein tertiary conformation. When exposed, aromatic amino acids such as tyrosine, tryptophan, and phenylalanine exhibit endogenous fluorescence, which is influenced by the conformation of the protein and its interaction with the surrounding environment. Tryptophan is particularly important among these aromatic amino acids due to its exceptional sensitivity and minimal interference, which enable accurate identification of positional changes in tryptophan residues within proteins and changes in protein structure. As depicted in Figure 7, tryptophan residues exhibit fluorescence within the wavelength range of 300–450 nm upon excitation at 280 nm. The intensity of tryptophan fluorescence is a quantitative indicator of the exposure level of tryptophan residues, with a higher intensity indicating enhanced exposure.

Compared with the fluorescence intensity of the control, that of the LWE samples decreased in a concentration-dependent manner with KCl addition (*p* < 0.05). This decrease in fluorescence intensity can be attributed to two factors. First, treatment with different concentrations of K^+^ and Na^+^ resulted in the expansion of LWE molecules, thereby exposing tryptophan and other chromophoric groups to a polar environment and leading to fluorescence quenching. Second, the addition of KCl and NaCl changed the microenvironment of tryptophan. This modification caused steric hindrance, resulting in weakened absorption of tryptophan and reduced intensity of the characteristic spectral peaks. This led to an increased degree of dissociation that is consistent with changes in size and group microenvironment. Additionally, salt exerted a protective effect on the stability of LWE proteins, thereby mitigating the increase in tryptophan levels and effectively inhibiting thermal protein aggregation. The observed decrease in fluorescence intensity indicated an increase in α-helix content and a decrease in β-sheet structure, indicating the inhibition of protein aggregation. Furthermore, the KCl treatment group exhibited a lower β-sheet content than the control group, supporting this conclusion and our analysis of secondary structure [36,37]. The maximum wavelength (λ_max_) primarily corresponds to the microenvironment of tryptophan residues. λ_max_ < 330 nm implies localization of tryptophan residues within a nonpolar region inside the protein molecule, while λ_max_ > 330 nm indicates the presence of tryptophan residues in a polar region on the outside of the protein molecule. In all the samples, λ_max_ exceeded 330 nm, indicating that the tryptophan residues were located in the external polar environment of the protein molecule. Compared to the control, the addition of KCl resulted in a λ_max_ closer to 330 nm and therefore a blueshift in the peak of endogenous fluorescence absorption. This implies that K^+^ and Na^+^ ions may induce the aggregation or recombination of exposed hydrophobic groups, leading to a more hydrophobic and less polar microenvironment surrounding tryptophan residues [19].

#### 3.6.2. Surface Hydrophobicity

**Figure 8:**
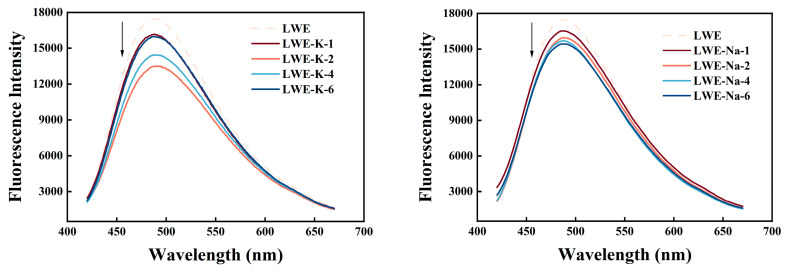
Effects of KCl and NaCl on the surface hydrophobicity of LWE.

The functional properties of proteins are influenced by surface hydrophobicity, which affects their interfacial properties. Hydrophobic interactions play a crucial role in maintaining protein stability, conformation, and function and are among the key forces involved in preserving protein tertiary structure [29]. As shown in Figure 8, the fluorescence intensity of the protein dispersions significantly increased after heat treatment in the absence of salt. However, the addition of KCl to LWE proteins led to a reduction in fluorescence intensity.

The observed decrease in fluorescence intensity in the protein dispersions containing salt can be attributed to the shielding effect of K^+^ charges on protein molecules, which results in encapsulation of the hydrophobic domain and decreases surface hydrophobicity [46]. Additionally, the binding of ANS with proteins is influenced by electrostatic interactions, leading to a reduction in fluorescence intensity. Previous studies have shown that hydrogen bonds are involved in protein aggregation induced by heat treatment, while salt can impede intermolecular hydrogen bond formation [36]. The addition of 2% KCl resulted in the lowest fluorescence intensity, indicating the least surface hydrophobicity (*p* < 0.05). Previous studies have demonstrated an inverse relationship between protein solubility and surface hydrophobicity. Therefore, reduced surface hydrophobicity may contribute to enhanced solubility. The surface hydrophobicity values in the KCl and NaCl treatment groups is consistent with the above solubility results. Surface hydrophobicity increased significantly upon the addition of a high concentration of KCl (*p* < 0.05). This enhancement can be attributed to the binding of K^+^ with proteins, leading to the concealment of surface hydrophobic groups at lower concentrations. However, at higher concentrations, excessive binding of K^+^ induces protein repulsion and dispersion, potentially exposing hydrophobic residues and further increasing surface hydrophobicity [22].

### 3.7. Relationships among Physical and Chemical Properties, Thermal Properties, and Structural Properties

**Figure 9:**
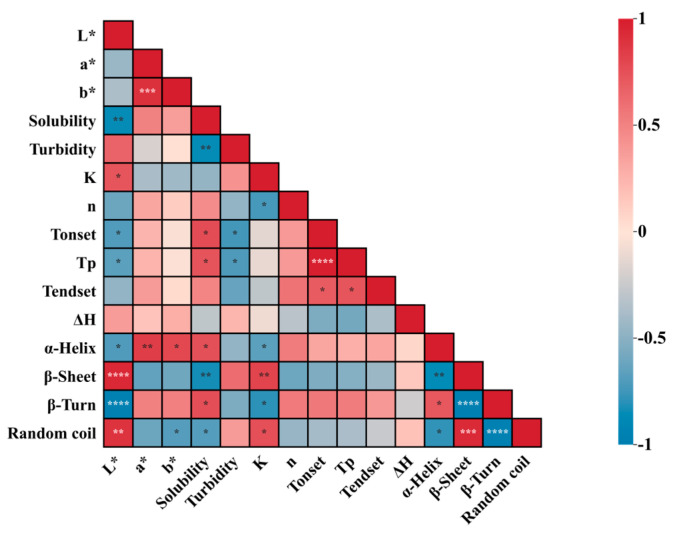
Relationships among the physical and chemical properties, thermal properties, and structural properties of LWE. * indicates significant, ** indicates extremely significant, *** indicates very significant, and **** indicates super significant.

As shown in Figure 9, the enhancement effect of salts on the thermal stability of LWEs was further investigated through correlation analysis to examine the interrelationships among physical and chemical properties, thermal properties, and structural properties.

The results demonstrated a robust correlation between *T*_p_ and *T*_onset_, as well as other properties; however, the correlation between *T*_endset_ and other properties was weak. Specifically, we observed a negative correlation between *T*_p_ and L*, while *T*_p_ was positively correlated with both a* and b*. These findings suggest that increasing *T*_p_ can effectively enhance the overall color of LWEs. Additionally, *T*_p_ exhibited a positive correlation with solubility, a negative correlation with turbidity, a weak negative correlation with K value, and a weak positive correlation with *n* value. These results suggest that *T*_p_ is influenced by these parameters. Increasing *T*_p_ results in decreased turbidity and apparent viscosity, increased solubility, and enhanced fluidity. This implies that enhancing the thermal stability of LWE can inhibit aggregation and improve rheological properties. *T*_p_ exhibited a positive correlation with α-helices and β-turns but a negative correlation with β-sheets and random coils. This result implies that enhancing the thermal stability of LWEs leads to an increase in α-helices and β-turns within the secondary structure, accompanied by a decrease in β-sheet and random coil contents. These changes hinder the transformation from α-helices and β-turns into β-sheets and random coils induced by thermal aggregation. Additionally, the change in Δ*H* exhibited a positive correlation with turbidity but negative correlations with solubility as well as with *T*_onset_, *T*_p_, and *T*_endset_. These findings suggest that enhancing the thermal stability of LWE reduces the energy required for network structure formation and delays gel network development. The present findings are consistent with previous results, demonstrating a robust correlation among thermal properties, physical and chemical properties, and structural properties. Furthermore, the ability of KCl to enhance the stability of LWEs is comparable to that of NaCl, suggesting its potential as a partial substitute for NaCl.

## 4. Conclusions

This study demonstrated that the addition of KCl has a significant impact on the thermal stability of LWE. Specifically, when 2% KCl was added, the solubility increased significantly, while the turbidity and particle size decreased, indicating the inhibition of heat-induced aggregation. The addition of different concentrations of KCl resulted in a significant decrease in *K* value and an increase in both the thermal transition temperature and T_p_. Structural analysis revealed an increase in α-helix content, a decrease in β-sheet content, and a downward shift in fluorescence intensity. Correlation analysis showed that T_p_ was positively correlated with solubility, α-helix structures, and β-turn structures but negatively correlated with turbidity, β-sheet structures, and random coil conformation. Overall, adding 2% KCl had similar effects as adding 2–6% NaCl on reducing heat sensitivity. Therefore, using KCl as a substitute for sodium salts in LWE products could improve their thermal stability while reducing their sodium content. These findings provide valuable insights for future research into sodium substitutes in LWE products.

## Figures and Tables

**Table 1:** Power law equation parameters for LWE samples with various KCl and NaCl concentrations. Different letters indicate significant differences (*p* < 0.05) among the data within each group.

Samples	Salt Concentration (%)	*K*/(Pa·s)	*n*	R^2^
LWE	0	0.490 ± 0.004 a	0.520 ± 0.007 e	0.99
LWE-K	1	0.156 ± 0.003 bc	0.756 ± 0.009 bc	0.97
	2	0.089 ± 0.003 cd	0.678 ± 0.014 cd	0.96
	4	0.227 ± 0.009 b	0.623 ± 0.020 d	0.95
	6	0.249 ± 0.010 b	0.661 ± 0.018 cd	0.95
LWE	0	0.406 ± 0.010 a	0.505 ± 0.021 e	0.98
LWE-Na	1	0.042 ± 0.000 d	0.565 ± 0.015 e	0.98
	2	0.023 ± 0.024 d	0.878 ± 0.004 b	0.98
	4	0.010 ±0.001 d	0.955 ± 0.002 a	0.96
	6	0.011 ± 0.001 d	0.974 ± 0.001 a	0.97

**Table 2:** Onset temperature (*T*_onset_), thermal denaturation peak temperature (*T*_P_), endset temperature (*T*_endset_), and denaturation enthalpy (Δ*H*) of LWEs with various KCl and NaCl concentrations. Different letters indicate significant differences (*p* < 0.05) among the data within each group.

Samples	Salt Concentration (%)	*T*_onset_/(°C)	*T*_p_/(°C)	*T*_endset_/(°C)	Δ*H*/(J/g)
LWE	0	78.017 ± 0.187 g	83.487 ± 0.133 g	90.280 ± 0.436 b	1.087 ± 0.046 abc
LWE-K	1	78.673 ± 0.116 f	83.940 ± 0.087 f	90.717 ± 0.421 b	1.250 ± 0.092 a
	2	79.770 ± 0.277 e	85.093 ± 0.029 e	90.893 ± 0.776 b	0.835 ± 0.126 c
	4	81.620 ± 0.128 c	86.233 ± 0.231 d	93.190 ± 0.543 a	1.009 ± 0.055 abc
	6	82.740 ± 0.122 b	87.553 ± 0.214 b	92.507 ± 1.243 ab	1.067 ± 0.147 abc
LWE-Na	1	78.650 ± 0.121 f	84.037 ± 0.219 f	90.867 ± 0.834 b	1.134 ± 0.132 ab
	2	80.203 ± 0.049 d	85.130 ± 0.104 e	90.617 ± 1.094 b	0.950 ± 0.089 bc
	4	82.697 ± 0.150 b	87.057 ± 0.196 c	90.543 ± 1.503 b	1.140 ± 0.15 ab
	6	83.460 ± 0.040 a	88.533 ± 0.040 a	93.257 ± 0.140 a	0.875 ± 0.031 bc

## Data Availability

The original contributions presented in the study are included in the article, further inquiries can be directed to the corresponding authors.
